# Woodland caribou calving fidelity: Spatial location, habitat, or both?

**DOI:** 10.1002/ece3.11480

**Published:** 2024-05-30

**Authors:** P. D. Walker, A. R. Rodgers, J. Shuter, J. M. Fryxell, E. H. Merrill

**Affiliations:** ^1^ Department of Biological Sciences University of Alberta Edmonton Alberta Canada; ^2^ Centre for Northern Forest Ecosystem Research Ontario Ministry of Natural Resources and Forestry Thunder Bay Ontario Canada; ^3^ Department of Integrative Biology University of Guelph Guelph Ontario Canada

**Keywords:** behavior, conservation, fitness, *Rangifer tarandus*, space use

## Abstract

Individuals that isolate themselves to give birth can use more than one strategy in choosing birth sites to maximize reproductive success. Previous research has focused on the consistency in the use of the same birth‐site across years (i.e., spatial fidelity), but individuals alternatively may use similar habitat conditions across years (i.e., habitat fidelity). Using GPS telemetry, we determined whether woodland caribou expressed spatial or habitat fidelity during calving, and evaluated intrinsic and extrinsic factors associated with expressing either type of fidelity. We identified 56 individuals with ≥2 putative birth events, via a movement‐based model, across northern Ontario between 2010 and 2014. Individuals were classified as expressing (1) spatial fidelity by comparing sequential calving locations to a random spatial distribution of available calving locations, (2) habitat fidelity using a logistic use model compared to a null (intercept only) model, (3) no fidelity (neither criterion met), or (4) both spatial and habitat fidelity (both criteria met). Across all individuals, 37% expressed no fidelity (36 of 98), 15% expressed only spatial fidelity (15 of 99), 35% expressed only habitat fidelity (34 of 98), and 14% expressed both spatial and habitat fidelity (14 of 98). Older individuals were more likely to express spatial fidelity, whereas lower availability of upland and lowland conifer forests without linear features increased the probability an individual expressed habitat fidelity. Our results indicate that managing for caribou calving needs to consider protecting both specific, known birthing sites, but also broad‐scale areas of preferred habitat for calving. Understanding the mechanisms that influence caribou expressing calving fidelity, and associated fitness costs, is crucial for the conservation of the species.

## Graphical abstract

Majority of woodland caribou expressed some type of fidelity during the calving period with habitat fidelity (50%) being more frequently expressed in calving sequences than spatial fidelity (29%). Older individuals were more likely to express spatial fidelity, whereas lower availability of high‐quality habitat increased the probability an individual expressed habitat fidelity. Our results indicate that protecting not only specific sites, but preferred calving habitat may be essential for enhancing opportunities for caribou calving.

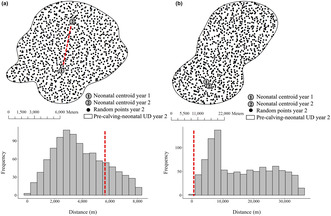

## 1 INTRODUCTION

The lifetime fitness of decision‐making animals is dependent on their behavioral response to trade‐offs between predation risk and resource acquisition (McNamara & Houston, 1986; Sih, 1980; Verdolin, 2006). To manage these trade‐offs during an annual cycle, mobile animals can choose to either move to new areas or continue to use previously used areas and express a form of interannual site fidelity (hereafter site fidelity; Greenwood, 1980, Switzer, 1993). Where site fidelity has been expressed, previous knowledge of available resources and predation risk at the site have been shown to increase an individual's fitness (Gavin & Bollinger, 1988; Gehr et al., 2020; Lafontaine et al., 2017; Welch et al., 2000). Site‐specific fidelity has been reported across a range of disparate animal taxa, including fish (Bunt et al., 2021; Compaire et al., 2022), birds (Gerber et al., 2019; Willie et al., 2020), amphibians (Balázs et al., 2020; Denoël et al., 2017), reptiles (Baltazar‐Soares et al., 2020; Evans et al., 2019), and mammals (Gehr et al., 2020; Morrison et al., 2021).

Site fidelity in previous studies has been understood almost exclusively as a spatial evaluation, regardless of the conditions at those locations (hereafter, referred to as spatial fidelity). For example, barren‐ground caribou (*Rangifer tarandus groenlandicus*) express spatial fidelity each spring as they return to traditional calving grounds, a defining characteristic of the subspecies, which is used to delineate local populations (Gunn & Miller, 1986). By returning to the same location, an individual likely benefits from the resources sufficient to support their life history requirements over time, and from the additional familiarity with them (Greenwood, 1980; Switzer, 1993; Wolf et al., 2009). Returning to a specific site has been attributed to an individual using spatial memory to relocate the site (Fagan et al., 2013; Lewis et al., 2021; Merkle et al., 2019). When an individual expresses spatial fidelity, it may be contingent on the spatial variability and predictability of habitat quality (Morrison et al., 2021; Switzer, 1993). For example, when spatial variability of habitat quality is low, i.e., most areas having similar value, spatial fidelity may be common because there is no benefit to expend more energy while exploring other sites (Switzer, 1993). In contrast, if habitat quality is highly variable across an area, a smaller extent of the area may provide high‐quality habitat (i.e., area‐heterogeneity trade‐off; Ben‐Hur & Kadmon, 2020; Kadmon & Allouche, 2007) and individuals may return to that area, but only if environmental conditions are predictable (i.e., autocorrelated through time; Morrison et al., 2021). In one of the few studies relating spatial fidelity to habitat availability, Chaverri et al. (2007) concluded tent‐making bats (*Artibeus watsoni*) in Costa Rica expressed higher spatial fidelity in regions with low roost availability, but they did not directly assess predictability of roost sites. If high‐quality sites fluctuate through time, it may be advantageous to not return to previously occupied sites (Teitelbaum & Mueller, 2019). For example, female red squirrels (*Sciurus vulgaris*) in Cumbria, England, expressed higher spatial fidelity for home ranges in areas where food resources did not fluctuate across time (Lurz et al., 1997).

As a second behavioral strategy, individuals could express “habitat fidelity”, i.e., interannual faithfulness to use a specific habitat during a time of year or life history event, irrespective of spatial location. Previous studies have evaluated consistency in habitat use across individuals (DeMars & Boutin, 2018; Gillingham & Parker, 2008; Merrill et al., 2020); however, these studies did not focus on consistency within individuals across years. An exception is the focus on behavior syndromes and individual personality traits, where studies have evaluated the interannual consistency of individuals selecting specific habitat characteristics over time (Hertel et al., 2019, 2020, 2021; Leclerc et al., 2016; Spiegel et al., 2017). In these few cases, the focus was on broad, seasonal time periods, not specific life history events. Expressing habitat fidelity during key life history events, in particular reproduction, may improve individual fitness, which we assume by definition does not rely on specific spatial memory of geographic locations. Natal habitat preference induction may result in individuals expressing habitat fidelity, where individuals are more likely to select habitats that they experienced early in life (Davis & Stamps, 2004; Larue et al., 2018). In the case of consistent use of nest or birth sites, the importance of spatial location versus habitat conditions for reproductive success has not been distinguished (Hoover, 2003; Lafontaine et al., 2017; Shields, 1984; Vergara et al., 2006). Understanding the fitness benefits of spatial fidelity vs. habitat fidelity or their combined effects during important life history events such as birthing, may be key in how we approach managing critical habitat, particularly for threatened or endangered species.

Complicating the assessment of an individual's motivation for fidelity is the possibility that advantages may also be contingent on endogenous factors, such as age and past experiences (Hoover, 2003; Shields, 1984; Switzer, 1993). Older individuals may express spatial fidelity because of greater experience, memory, or knowledge about habitat cues, or because they tend to invest more in reproduction as they age (terminal investment hypothesis, Williams, 1966). In contrast, the “win‐stay: lose‐switch” strategy suggests that if an individual is reproductively successful (i.e., wins), then it may be beneficial to express spatial fidelity in the following year, but not if the offspring dies (i.e., loses, Switzer, 1993). For prothonotary warblers (*Protonotaria citrea*) in Illinois, USA, Hoover (2003) showed that >80% of individuals returned to the same site when they produced two broods in the previous year, whereas <60% of individuals returned to the same site if they only produced one brood or none at all. We suggest that age and reproductive success is also likely to promote habitat fidelity, but whether they influence spatial or habitat fidelity more is unknown.

In this paper, we investigated whether boreal woodland caribou (*R. t. caribou*, hereafter caribou) express interannual spatial and/or habitat fidelity in northern Ontario, Canada, during a crucial period for reproductive success (Gustine et al., 2006; Pinard et al., 2012; Walker et al., 2021) that can influence population dynamics in this threatened species (Decesare et al., 2012; Gaillard et al., 2000). Previous studies have documented interannual spatial fidelity for caribou during the calving period but did not assess the alternative habitat‐fidelity strategy (Faille et al., 2010; Lafontaine et al., 2017; Schaefer et al., 2000; Silva et al., 2020; Wittmer et al., 2006). Although individual caribou during calving have shown some consistency in selecting for shorelines, lowlands, and mid‐late seral (≥20 years) stands of conifers (Carr et al., 2007; Hornseth & Rempel, 2016; Pinard et al., 2012; Viejou et al., 2018; Walker et al., 2021), none of these studies distinguished between habitat and spatial fidelity, and the conditions under which caribou may exhibit these different forms of fidelity.

We predicted caribou would exhibit spatial fidelity when high‐quality habitat was limited and did not change over time (i.e., predictable). Consistent with foraging theory, we expected individuals would specialize on preferred habitats when they are abundant because search time is low (Charnov, 1976; MacArthur & Pianka, 1966). Therefore, we predicted caribou would express habitat fidelity when high‐quality habitat was readily available but not predictable. No fidelity would be expressed when high‐quality habitat was limited and unpredictable, whereas simultaneous spatial and habitat fidelity would be observed when high‐quality habitat was predictable and available. We also expected older caribou whose calves survived the previous year to express fidelity, because of greater experience with age and the “win‐stay: lose‐switch” strategy (Switzer, 1993). Identifying whether caribou rely on specific sites or habitat conditions for successful reproduction is key for the management of this species because they are listed as threatened under Canada's *Species at Risk Act* (Government of Canada, 2019) and Ontario's *Endangered Species Act* (Government of Ontario, 2007).

## 2 STUDY AREA

**FIGURE 1:**
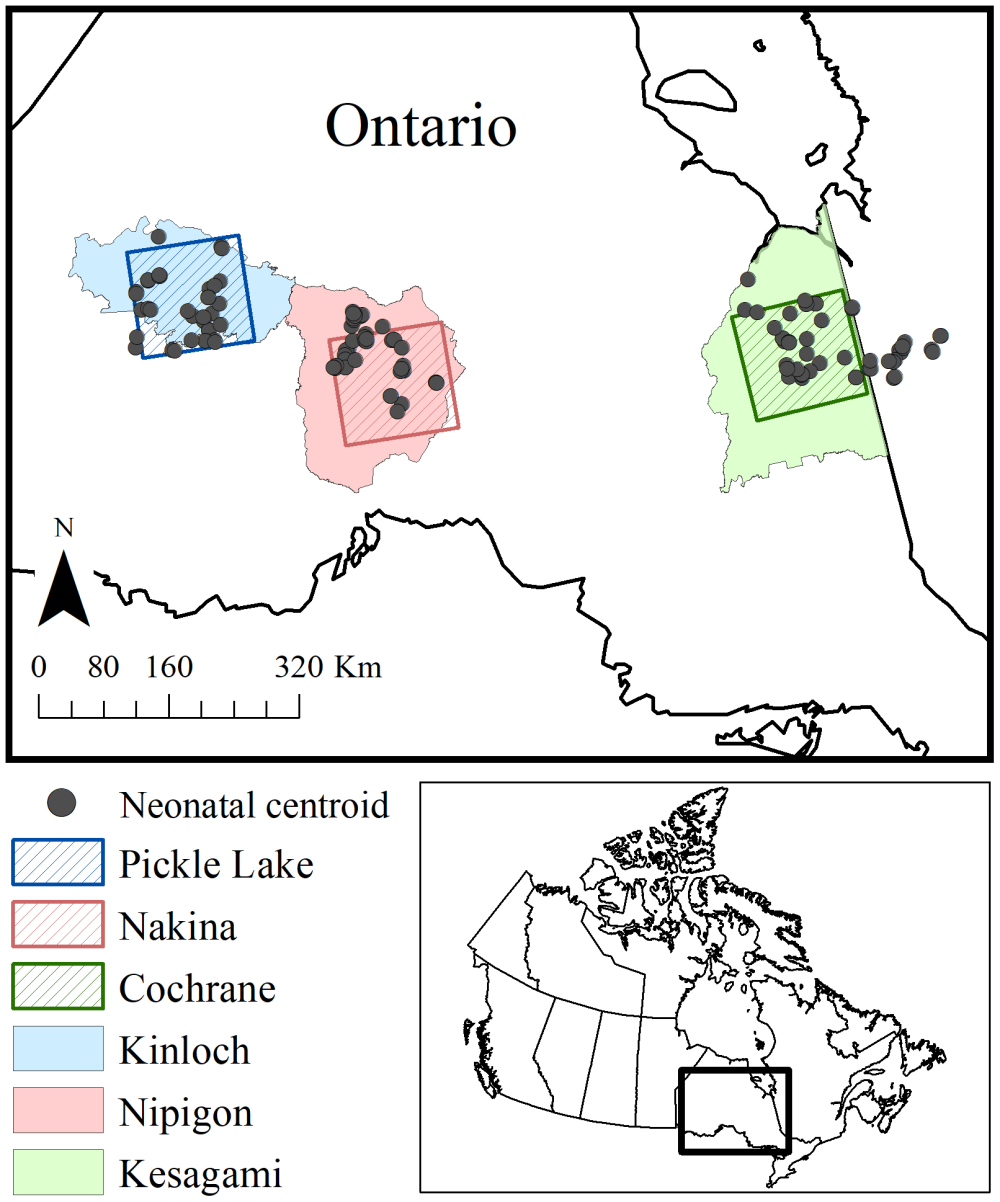
Location of neonatal centroids across the three study regions (Pickle Lake, Nakina, and Cochrane) within northern Ontario, Canada, 2010‐2014, and their respective designated local caribou populations (Kinloch, Nipigon, and Kesagami).

We evaluated calving fidelity across northern Ontario, Canada, within three study regions: Pickle Lake (90.938 W, 51.568 N; 23,000 km^2^), Nakina (87.548 W, 50.388 N; 23,000 km^2^), and Cochrane (80.598 W, 49.908 N; 23,000 km^2^; Figure 1). All regions were within the boreal zone, which is characterized by stands of black spruce (*Picea mariana*), jack pine (*Pinus banksiana*), balsam fir (*Abies balsamea*) trembling aspen (*Populus tremuloides*), and white birch (*Betula papyrifera*) (Rowe, 1972). Pickle Lake and Nakina are located within the Boreal Shield of northwestern Ontario, which is dominated by rolling topography, whereas Cochrane is in the Northern Clay Belt region of northeastern Ontario and characterized by minimal topographical variation (McMullin et al., 2013; Thompson et al., 2015). Consequently, Cochrane had the highest extent of lowland conifer forest (swamp, bog, and fen; 64%), whereas Nakina and Pickle Lake both had a lower extent at 28% (Walker et al., 2021). Disturbance regimes differed across the three study regions. At the time of the study (2010), Nakina had greater amounts of post‐harvest regeneration (<40 years; 22%) compared to Cochrane (13%) or Pickle Lake (0.04%, Fryxell et al., 2020; OMNRF, 2014a), whereas Pickle Lake had a higher proportion of natural disturbance (proportion burned <50 years; 12%) compared to Nakina and Cochrane (both 4%, Fryxell et al., 2020; OMNRF, 2014a). Linear feature density was higher in Nakina (0.42 km/km^2^) and Cochrane (0.31 km/km^2^), compared to Pickle Lake (0.05 km/km^2^, Fryxell et al., 2020; OMNRF, 2014a). Wolf (*Canis lupus*) and moose (*Alces alces*) densities were highest in Nakina (6.7 wolves/1000 km^2^, 11.8 moose/100 km^2^) with lower densities in Pickle Lake (4.2 wolves/1000 km^2^, 4.6 moose/100 km^2^) and Cochrane (3.7 wolves/1000 km^2^, 3.8 moose/100 km^2^, Fryxell et al., 2020; OMNRF, 2014a), whereas black bear (*Ursus americanus*) densities were similar across all regions (20–40 black bears/100 km^2^, Howe et al., 2013; Rodgers et al., 2009). Total annual precipitation was greater in Cochrane (824 ± 81 mm; $\bar{x}$ ± SD, 20‐year average [1991–2010]) than Nakina (776 ± 130 mm) and Pickle Lake (736 ± 122 mm; Environment Canada, https://climate.weather.gc.ca/historical_data/search_historic_data_e.html, accessed 14 Jun 2019). January daily temperatures were highest in Cochrane (−17.82 ± 3.69°C), followed by Nakina (−18.60 ± 3.61°C) and Pickle Lake (−19.26 ± 3.60°C), however July daily temperatures were similar among areas (Pickle Lake: 17.66 ± 1.49°C; Nakina: 17.07 ± 1.50°C; Cochrane: 17.36 ± 1.25°C).

## 3 METHODS

### 3.1 Caribou capture, monitoring, and ranges

We captured 166 female caribou in Pickle Lake (PL; *n* = 52), Nakina (NA; *n* = 58), and Cochrane (CO; *n* = 56) between 2010 and 2014 using a net gun and fitted them with either GPS‐Argos (Telonics Inc., Mesa, AZ, USA; Lotek Wireless Inc., Newmarket, ON, Canada) or GPS‐Iridium radio collars (Lotek Wireless Inc., Newmarket, ON, Canada). Animal capture and handling protocols were approved by the Ontario Ministry of Natural Resources and Forestry Wildlife Animal Care Committee (protocols 10‐183, 11‐183, 12‐183, 13‐183, and 14‐183). Caribou age at capture was estimated using tooth eruption and wear (van den Berg et al., 2021). For caribou, van den Berg et al. (2021) found that experienced and unexperienced blind observers were able to correct estimate age via tooth wear and eruption within a 2‐year deviation of the “true” age (via cementum annuli) 83% and 85% of the time, respectively. Caribou GPS fixes were obtained every 1, 2.5, 5, or 12.5 hrs for multiple years resulting in 282 caribou‐years. Mean fix rate success across all 282 caribou‐years was 97% (range: 90–100%). All caribou were non‐migratory, i.e., sedentary woodland caribou (sensu Pond et al., 2016).

We identified annual parturition events following the individual‐based model of DeMars et al. (2013) based on a reduction in caribou movement rates. DeMars et al. (2013) found a 97.5% accuracy when using a 4‐h fix rate interval to predict parturition events, whereas using the same approach, Walker et al. (2021) found a 100% accuracy when using 2.5‐ or 3‐h fix rate intervals. Because fix rate intervals of caribou in this study were more variable, we first evaluated the sensitivity of predicting parturition events with different fix rate intervals (1, 2.5, 5 or 12.5 h) compared to parturition events identified from video footage of 20 caribou (22 caribou‐years, PL: *n* = 4, NA: *n* = 9, CO: *n* = 9) equipped with animal‐borne video collars (Thompson et al., 2012; Viejou et al., 2018; Walker et al., 2021). We found accuracy levels of 91% for predicting parturition using fix rate intervals up to 12.5‐h with zero false positives (i.e., a parturition event is predicted when one did not occur, Data S1). Therefore, we applied the individual‐based model of DeMars et al. (2013) to 260 of the 282 caribou‐years (PL: *n* = 83, NA: *n* = 91, CO: *n* = 86) with fix rate intervals between 1‐ and 12.5‐h and unknown birth status (i.e., did not have animal‐borne video collars).

**FIGURE 2:**
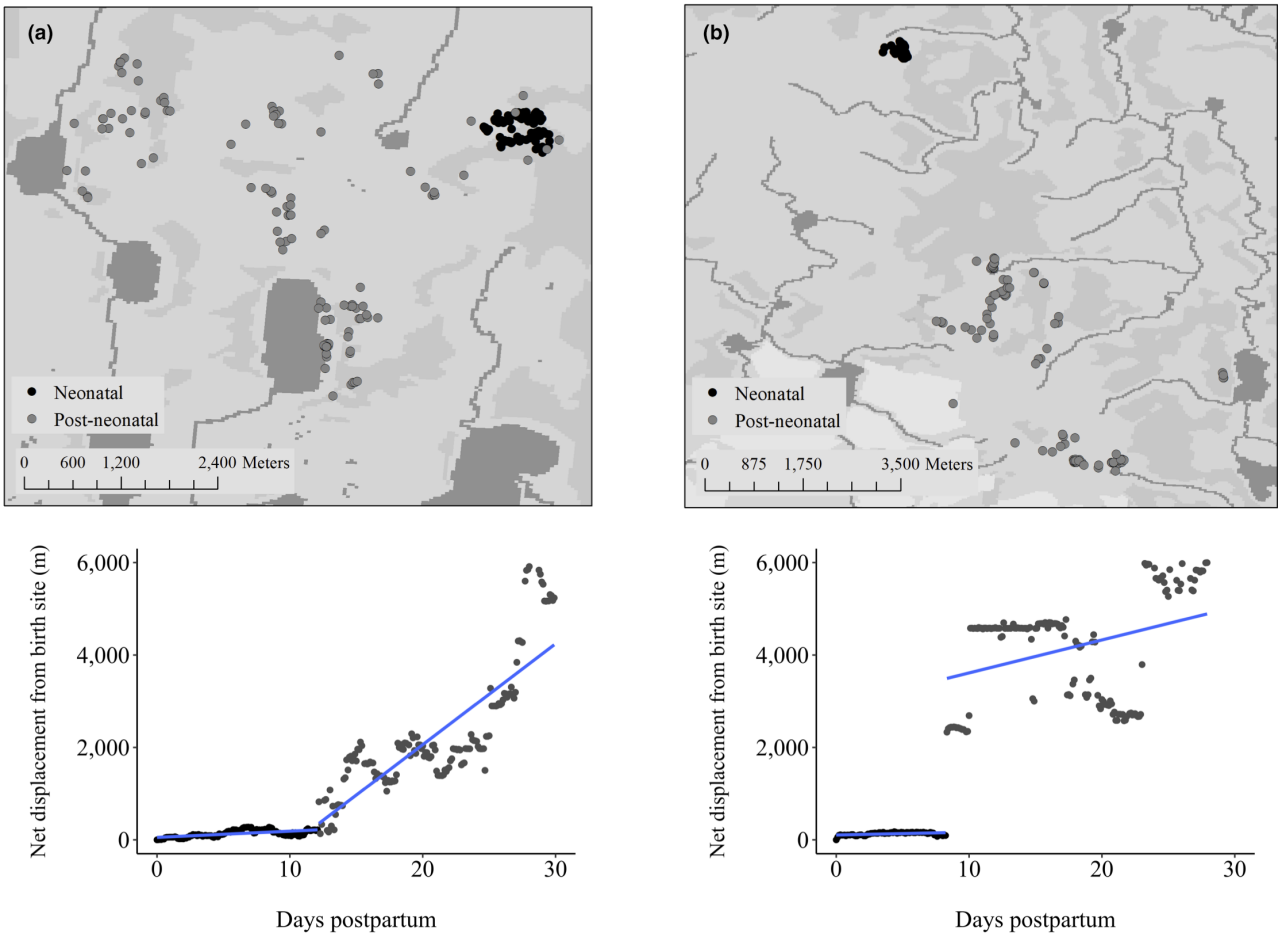
Illustration of the approach to identify habitat fidelity. We first define the neonatal locations for a calving‐sequence in (a) year one and (b) year two by relating the rate of net displacement (m) from the parturition site to all subsequent relocations postpartum, where we fit a piece‐wise regression to determine the breakpoint in the net displacement after parturition. Once the neonatal locations are determined, we identify the Far North Land Cover types used in year one and two and fit a logistic model, compared to a null model (intercept only), to identify if a calving‐sequence expresses habitat fidelity or not. This approach was applied to 98 calving‐sequences across northern Ontario, Canada, 2010–2014.

From the 166 caribou, we identified 56 individuals (133 caribou‐years) with ≥2 known (via video collar footage) or predicted birth events and used GPS locations from these individuals to define calving‐sequences. We derived a pre‐calving‐neonatal range for each caribou‐year in time *t*, based on the 95% isopleth utilization distribution (UD) using the reference bandwidth for smoothing (Walker et al., 2021) for the 30‐day period prior to parturition (i.e., pre‐calving), and a variable number of days neonatal period, based on a breakpoint in postpartum displacement from the birth site (i.e., neonatal locations, Figure 2; see details in Walker et al., 2021). To standardize fix‐rate sampling for 95% UDs, caribou with 1‐h fix rates were randomly resampled to 13‐h, caribou with 2.5‐h fix rates to 12.5‐h, and 5‐h fix rates to 15‐h to make sampling more comparable across caribou‐years. A calving‐sequence was determined from the same caribou in year *t* compared to year *t* + 1 for up to 3 years. For example, the centroid (i.e., geometric mean) and habitat composition of the neonatal locations in year *t* were compared to those in year *t* + 1 and *t* + 2, and in year *t* + 2 to those in *t* + 3. In the final analysis, we used 99 calving‐sequences to evaluate spatial fidelity but only 98 sequences to evaluate habitat fidelity (see below).

### 3.2 Spatial and habitat fidelity

**FIGURE 3:**
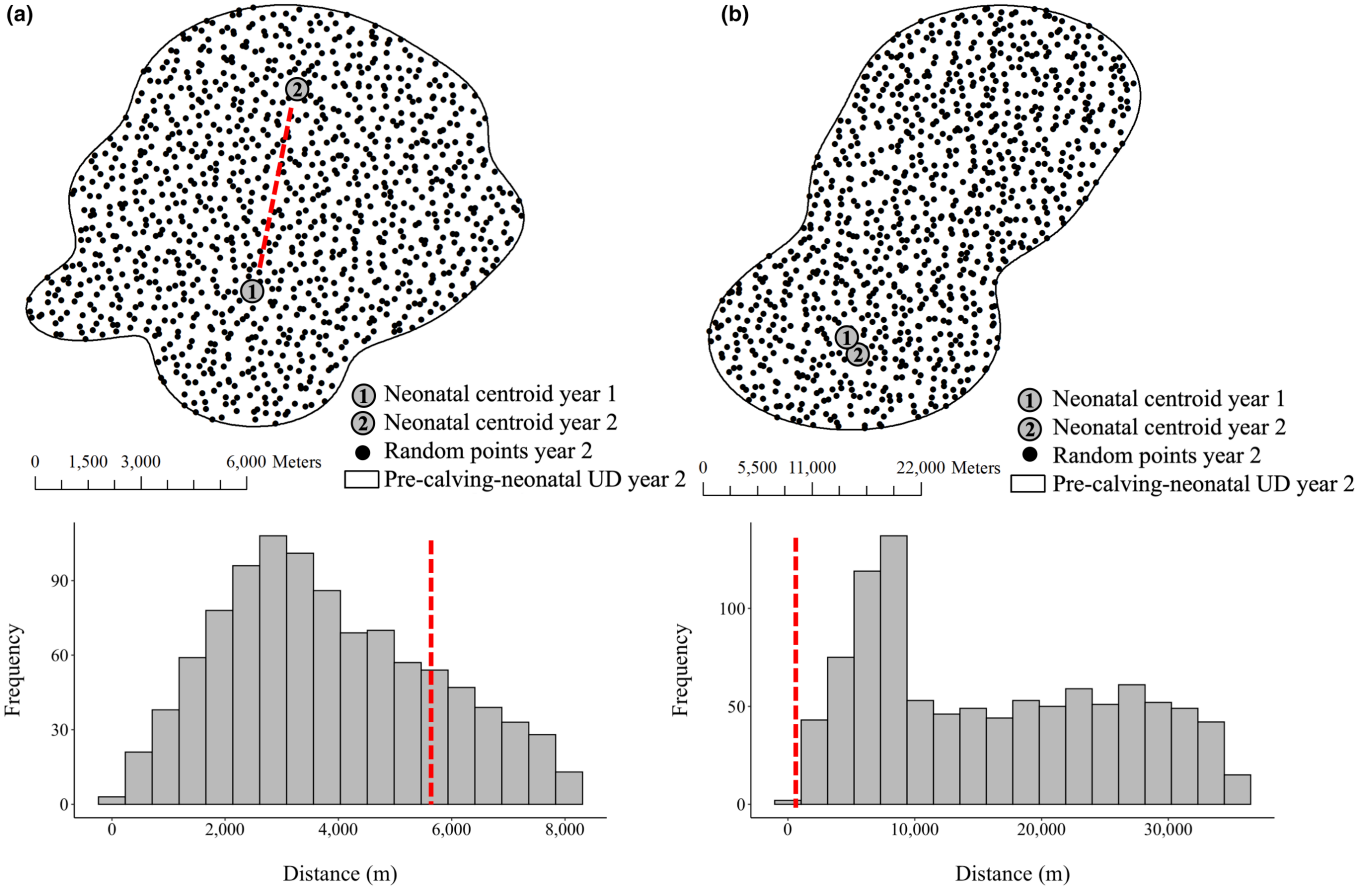
Illustration of how a calving‐sequence was determined to reflect spatial fidelity, by calculating the proportion of random distances of where the individual could have calved in the following year that are less than or equal to the distance between the neonatal centroid in year one and year two. The distribution of random distances is derived by calculating the distance between the neonatal centroid in year one and the 1000 random locations within the pre‐calving‐neonatal utilization distribution (UD) in year two. If the proportion of random distances that is less than or equal to the distance between neonatal centroids is (a) greater than 0.05 (e.g., 0.68), then we conclude that the calving‐sequence did not reflect spatial fidelity. Alternatively, if the proportion of random distances less than or equal to the distance between neonatal centroids is (b) smaller than 0.05 (e.g., 0.001), then we conclude that the calving‐sequence reflected spatial fidelity. This approach was applied to 99 calving‐sequence across northern Ontario, Canada, 2010–2014.

We assessed spatial and habitat fidelity in calving‐sequences, and by default, we defined no fidelity to occur when we had no evidence of neither spatial nor habitat fidelity. We assessed the spatial fidelity of each calving‐sequence by comparing the Euclidian distance (m) between the observed two neonatal centroids in sequential years to an expected distribution of distances (null) based on the distance between the neonatal centroid in the first year of a sequence and 1000 random locations generated within the pre‐calving‐neonatal range in the second year (Figure 3). The distribution of 1000 random distances produced the expected (i.e., null) distribution of distances reflecting where the individual could have calved in the second year given the size and shape of the pre‐calving‐neonatal range. We then calculated the proportion of random distances that were less than the distance between the two neonatal centroids and concluded a calving‐sequence expressed spatial fidelity if that proportion was less than 0.05. We used a threshold value of 0.05 to distinguish spatial fidelity based on preliminary analysis, because the variation in distances between neonatal centroids was substantially greater with values >0.05 (Data S1). We used a one‐tailed Mann–Whitney *U* test to evaluate if the distances between observed centroids of calving‐sequence designated as expressing spatial fidelity were significantly less than calving‐sequences that did not.

We evaluated habitat fidelity by comparing the interannual consistency in use of four land cover types at the neonatal GPS locations in the first (value of 0) and second year (value of 1) in logistic regression and used model selection based on Akaike's Information Criterion corrected for small sample size (AIC_c_, Burnham & Anderson, 2002). We concluded a calving‐sequence expressed habitat fidelity if the model including land cover types, as a single categorical variable, was ΔAIC_c_ < 4 from the null model, i.e., no difference in the land cover types used in the first and second year when compared to the null model. In contrast, a ΔAIC_c_ ≥ 4 indicates substantial differences in the land cover types used in the first and second year compared to the null model; therefore, we concluded the calving‐sequence did not express habitat fidelity. We recognize habitat reflects a multi‐dimensionality of abiotic and biotic factors specific to a species (Hall et al., 1997; Hutchinson, 1957), but given limited data on habitat covariates, we describe habitat fidelity based on caribou response to four land cover types and habitat quality (defined below) based on caribou response to these land cover types and linear anthropogenic features.

The four land cover types were lowland conifer forest (bog, swamp, or fen), upland conifer forest (coniferous and sparse), mixed‐deciduous forest (mixedwood and deciduous), and early seral forest (<20 years old), which were collapsed from 24 land cover types in the Ontario Far North Land Cover layer (version 1.4, OMNRF, 2014a). We focused on these land cover types because Walker et al. (2021) showed that caribou selected for lowland conifer forest and selected against early‐seral forest during calving, and others in these study regions showed throughout the year caribou selected against mixed‐deciduous and early seral forests, which reflect high risk areas due to high wolf use (Avgar et al., 2015; Hornseth & Rempel, 2016; Kittle et al., 2015, 2017; McGreer et al., 2015; Viejou et al., 2018). We also used these generalized land cover types to mitigate inaccuracies in finer land cover classification. Land cover was updated annually to account for annual disturbance created through forest fires (Aviation, Forest Fire and Emergency Services Fire Disturbance Area layer) and silvicultural practices based on Ontario Ministry of Natural Resources and Forestry (OMNRF) depletion records (OMNRF, unpublished data). We removed one calving‐sequence from the habitat fidelity analysis because the neonatal locations of the calving‐sequence were outside the spatial extent of the Far North Land Cover layer.

### 3.3 Factors influencing types of fidelity

We classified each calving‐sequence to four fidelity classes: no fidelity (neither spatial nor habitat fidelity), spatial fidelity, habitat fidelity, or both spatial and habitat fidelity. We then compared each fidelity type (1) to not expressing that specific fidelity type (0) as a function of intrinsic (age and calf survival) and extrinsic factors (habitat quality and predictability) that are described below. Because the four fidelity classes were not mutually exclusive, i.e., a calving‐sequence classified as expressing both spatial and habitat fidelity was also classified as expressing spatial fidelity as well as habitat fidelity, we used independent, binomial comparisons of each fidelity class instead of a multinomial model to distinguish among the four classes. We conducted three analyses based on different datasets. Because age and calf survival were known for only a subset of the caribou, we first used data from all caribou (55 caribou; 98 calving‐sequences) to assess the association between proportion of neonatal locations within each land cover type (fitting separate models for each land cover type), and habitat quality and predictability (see below) within the pre‐calving‐neonatal 95% UD with each type of fidelity. We used a mixed‐effects logistic regression with a random intercept for caribou ID because there was more than 1 calving‐sequence for 19% of caribou (range: 1–6 sequences per caribou). Second, for a subset of 41 caribou (70 calving‐sequences) with known ages, we assessed the association between age and the probability an individual caribou expressed each fidelity class using the same modeling approach as above. Given the possible imprecision in estimating age via tooth wear and eruption, we ran a sensitivity analysis on models that indicated a significant effect of age on expressing a type of fidelity. Specifically, we induced error in our estimates of age by changing each age by a random integer between 0 and 2, reran the mixed‐effect logistic model 100‐times, and reported the mean *p*‐value and 95% CIs. We randomly changed caribou ages between 0 and 2 years, because van den Berg et al. (2021) found observers correctly estimated age via tooth wear and eruption within a 2‐year deviation of age determined from cementum annuli 83–85% of the time. Finally, we used a subset of 27 caribou (27 caribou‐sequences) when neonatal survival (within 5‐weeks postpartum) during the first year was known to assess the association between a type of fidelity and calf survival in the previous year, and did not include a random intercept for caribou ID because there was only one calving‐sequence per caribou. Based on Walker et al. (2021), neonatal survival was predicted with an accuracy of 88% for the 5‐weeks postpartum using movement patterns of caribou with calves when compared to video footage of 20 caribou with calves (22 caribou‐years).

Habitat quality was based on the values predicted from a resource selection function (RSF, Manly et al., 2002) derived by pooling data on female, GPS‐collared caribou across all three regions. Used locations were the neonatal locations (defined above) of the female GPS‐collared caribou for which we determined calving‐sequences, with a median of 52 locations/caribou‐year collected between 7 May and 13 June. Available locations were the 1000 random locations within the pre‐calving‐neonatal home range of each caribou‐year that were used to classify spatial fidelity. We derived the RSF using the same four landcover classes as above and the density of linear anthropogenic features (km/km^2^), which included roads, powerlines, and railways (Hornseth & Rempel, 2016; Walker et al., 2021). We focused on these features because caribou showed strong selection for or against them during calving (Hornseth & Rempel, 2016; Viejou et al., 2018; Walker et al., 2021). Covariate values were assigned at each used and available location based on the 30‐m pixel in which a location fell. We used a mixed‐effect, logistic regression to estimate the parameters of an exponential RSF and included caribou‐year as a random intercept (Gillies et al., 2006). We used AIC_c_ and a threshold of ΔAIC_c_ < 4 to identify the top RSF model (Burnham & Anderson, 2002). We used k‐fold cross‐validation withholding 20% of the caribou‐years to evaluate the performance of the top RSF based on five folds (Boyce et al., 2002).

The top model was used to predict RSF values across all three regions at the 30‐m pixel scale. A metric of habitat quality was derived for each of the pre‐calving‐neonatal ranges during the first year of the calving‐sequence based on the mean RSF value within the extent of the range. Habitat predictability reflected the change in habitat quality between years due to forest disturbances from forest fires and silvicultural practices. Habitat predictability was calculated as the difference in the mean habitat quality (RSF_*t*_ − RSF_*t*+1_) of the first‐ and second‐year pre‐calving‐neonatal range. High predictability (similar habitat quality between years) was denoted by values approaching zero, whereas large values reflected low predictability (a large difference in habitat quality between years). All statistical analyses were conducted in the statistical computing program R (version 4.2.1, R Core Team, 2022).

## 4 RESULTS

### 4.1 Spatial and habitat fidelity

We classified one calving‐sequence as expressing spatial fidelity that was statistically identified as not expressing spatial fidelity (centroids were 1059 m apart, *p* = .14) because it was consistent with spatial fidelity found in the two other calving‐sequences from that individual in other years (Data S1). We classified a second calving‐sequence as expressing spatial fidelity because the distance between centroids (424 m, *p* = .07) was less than half the mean distances (1068 m) of all other calving‐sequences classified as expressing spatial fidelity (Data S1). Sizes of pre‐calving‐neonatal ranges during the first year (958 ± 1657 km^2^, ±SD, *n* = 99) did not differ among caribou expressing the four fidelity classes (*t*‐test, *p* > .33). The mean age of caribou with calves that survived (6.5 ± 2.1 years, ±SD, *n* = 16) and calves that did not survive (5.5 ± 0.6, *n* = 5) did not differ (*t*‐test, *p* = .11).

Of the 99 calving‐sequences, 36% exhibited no fidelity (*n* = 36), 29% exhibited spatial fidelity (*n* = 29), 50% exhibited habitat fidelity (*n* = 49), and 14% exhibited both habitat and spatial fidelity (*n* = 13; 98 calving‐sequences were used to calculate proportions expressing no fidelity, habitat fidelity, and both spatial and habitat fidelity; fidelity types were not mutually exclusive). The distances between neonatal centroids were significantly less for calving‐sequences that were classified as expressing spatial fidelity (1046 ± 1245 m, $\bar{x}$ ± SD) than calving‐sequences classified as not expressing spatial fidelity (15,735 ± 16,307 m, *p* < .001).

The proportion of caribou neonatal locations for calving‐sequences expressing habitat fidelity was higher in lowland conifer forest (0.74 ± 0.37) than upland conifer forest (0.22 ± 0.35), early seral forest (0.03 ± 0.14), and mixed‐deciduous forest (0.003 ± 0.01). Twenty and 31% of calving‐sequences had a proportion of their neonatal locations within mixed‐deciduous (20 of 98) and early seral forests (30 of 98), respectively, whereas 97 and 79% had neonatal locations within lowland conifer (95 of 98) and upland conifer forests (77 of 98), respectively. Caribou expressing habitat fidelity had a greater proportion of neonatal locations in lowland conifer forest and a lower proportion of neonatal locations in upland conifer forest compared to those not expressing habitat fidelity (Table 1). Greater proportion of neonatal locations in mixed‐deciduous forests was associated with expressing no fidelity and lower proportion of locations in mixed‐deciduous forests was associated with expressing habitat fidelity (Table 1). There was no effect of proportion of neonatal location in early seral forests expressing any type of fidelity.

**TABLE 1:** Number (*n*) of calving‐sequences, beta coefficient (*β*) and 95% confidence intervals (CI) from independent, logistic mixed‐effect models estimating the probability of caribou calving‐sequences expressing a type of fidelity (1: neither spatial nor habitat fidelity, spatial fidelity, habitat fidelity, or both spatial and habitat fidelity) compared to not expressing that type of fidelity (0) as a function of proportion of neonatal locations within upland conifer forests, lowlands conifer forests, early‐seral forests, or mixed‐deciduous forests, as a univariate covariate, across three study regions in northern Ontario, Canada, based on caribou telemetry data from 2010 to 2014.

Fidelity type	*n*	Upland conifer		Lowland conifer		Early‐seral		Mixed‐deciduous	
		*β*	95% CI	*β*	95% CI	*β*	95% CI	*β*	95% CI
Neither	98	1.07	−0.44, 2.58	−1.36	−2.78, 0.05	1.06	−2.70, 4.82	44.90*	9.77, 78.86
Spatial	98	1.36	−0.37, 3.08	−0.77	−2.34, 0.79	−2.67	−9.25, 3.92	−77.65	−169.92, 28.28
Habitat	98	−3.11*	−6.01, −0.20	3.12*	0.28, 5.93	−0.01	−5.49, 5.47	−43.46*	−84.79, −2.13
Both	98	−3.89	−14.24, 6.47	0.14	−5.53, 12.68	−1.24	−21.58, 19.11	−50.57	−347.77, 246.63

*Note*: Asterix indicates confidence intervals do not overlap zero.

### 4.2 Habitat quality and predictability

The top RSF indicated that during the neonatal period, caribou selected more strongly for areas that were lowland conifer and upland conifer forests than early seral and mixed‐deciduous forests, and areas with lower densities of linear features (Table 2). There was considerable support for the top model (model weight = 1.00; ΔAIC_c_ of 452 to the next candidate model) with a k‐fold cross‐validation score of 0.80. There was no difference in mean predicted RSF values of the pre‐calving‐neonatal ranges between years within calving‐sequences (*p* = .58, paired *t*‐test), with 92% of calving‐sequences having no change in habitat quality ($\bar{x}$ predictability = 0.0003 ± 0.002, ±SD, range: 0.00–0.02). Predictability values were similarly low at random points across all three study regions (Pickle Lake: 0.001 ± 0.002, *n* = 100,000; Nakina: 0.0002 ± 0.0001, *n* = 100,000; Cochrane 0.0009 ± 0.0001, *n* = 100,000). As a result, models evaluating the influence of predictability on the fidelity of caribou did not converge.

**TABLE 2:** Model coefficients, number of model parameters (*K*), Akaike's Information Criterion corrected for small sample size (AIC_c_), change in AIC_c_ from the best model (ΔAIC_c_), and model weights calculated from AIC_c_ (*w*_*i*_) for competing resource selection functions relating the relative probability of selection to land cover classes (LC; upland conifer forest, early seral forest, and mixed‐deciduous [dec.] forest) in reference to lowland conifer forest and to linear feature density (LF; km/km^2^) derived from caribou neonatal locations from 98 calving‐sequences and random locations in pre‐calving‐neonatal ranges across three study regions in northern Ontario, Canada, 2010–2014.

Model	Upland conifer	Early seral	Mixed‐dec.	LF	*K*	AIC_c_	ΔAIC_c_	*w*_*i*_
LC + LF	0.23	−0.56	−1.34	−0.85	5	57,984.08	0.00	1.00
LC	0.23	−0.81	−1.37		4	58,435.82	451.74	0.00
LF				−0.98	2	58,616.85	632.77	0.00
Null					1	59,299.55	1315.47	0.00

### 4.3 Factors influencing types of neonatal fidelity

In univariate models for each fidelity type, we found only individual age and habitat quality influenced whether caribou expressed spatial fidelity or habitat fidelity, respectively (Table 3). The probability of caribou expressing habitat fidelity increased as mean RSF value within the pre‐calving‐neonatal ranges decreased. Individuals were more likely to express spatial fidelity with increased age (Table 3), with caribou expressing spatial fidelity averaging 1.5 years older (7.05 ± 2.40, ±SD, *n* = 22) than caribou that did not (5.55 ± 1.61, *n* = 48). Based on our sensitivity analysis of inducing error in the estimates of age, individuals remained more likely to express spatial fidelity with increased age (0.04 ± 0.008, $\bar{x}$ *p*‐value ±95% CI). When we included habitat quality and age in a multivariable model, the habitat quality x age interaction was not statistically significant (Data S1). Finally, we found no evidence among our sample of 27 females that the survival of a calf in the previous year influenced probability of a caribou expressing any type of fidelity (Table 3).

**TABLE 3:** Number (*n*) of calving‐sequences, beta coefficients (*β*), and 95% confidence intervals (CI) for mean habitat quality in the pre‐calving‐neonatal 95% utilization distribution, caribou age (years), or calf survival (0/1) within the first 5‐weeks postpartum based on four independent, mixed‐effect, logistic models predicting the probability of a caribou calving‐sequences expressing a type of fidelity (1: neither spatial nor habitat fidelity, spatial fidelity, habitat fidelity, or both spatial and habitat fidelity) compared to not expressing that type of fidelity (0) based on caribou telemetry data from 2010 to 2014 across three study regions in northern Ontario, Canada.

Fidelity type	Habitat quality			Age			Calf survival		
	*n*	*β*	95% CI	*n*	*β*	95% CI	*n*	*β*	95% CI
Neither	98	3.14	−0.21, 6.49	70	−0.50	−1.03, 0.03	27	0.12	−1.62, 1.86
Spatial	98	0.29	−3.04, 3.62	70	0.47*	0.08, 0.86	27	−0.70	−3.11, 1.73
Habitat	98	−7.66*	−13.54, −1.77	69	−0.0007	−0.50, 0.44	27	0.69	−1.08, 2.46
Both	98	−12.11	−28.59, 4.36	69	−0.02	−0.96, 0.92	27	1.21	−14.86, 17.28

*Note*: Asterisk indicates confidence intervals do not overlap zero.

## 5 DISCUSSION

### 5.1 Spatial fidelity

We found the majority (~ 60%) of caribou expressed some type of fidelity during the neonatal period with habitat fidelity (50%) being more frequently expressed than spatial fidelity (29%). The proportion of caribou expressing interannual spatial fidelity is similar to the 25% of moose in Ontario, Canada (Welch et al., 2000), lower than the 64% of migratory caribou in Quebec, Canada (Brown et al., 1986), and higher than the 18% reported for pronghorn (*Antilocapra americana*) in Montana, USA (Wiseman et al., 2006). However, comparisons across studies can be difficult due to the different criteria used. A key component of detecting spatial fidelity in this study was the comparison to a random expectation to control for the possibility of spatial fidelity emerging randomly due to an individual's inherent movements (Picardi et al., 2023). The comparison to a random expectation has not always been used in past spatial fidelity studies. Instead, studies have used a subjective, a priori value to conclude if an individual animal expressed spatial fidelity (Brown et al., 1986; Welch et al., 2000; Wiseman et al., 2006). For example, Wiseman et al. (2006) assumed a priori if sequential birth sites were within 1 km of each other for individual pronghorn in Montana, USA, then they expressed spatial fidelity. Previous studies have also considered spatial fidelity along a behavioral gradient instead of a binary behavior (Faille et al. 2010, Morrison et al., 2021), which again can make comparisons difficult across studies and may result in different conclusions. In contrast, Rettie and Messier (2001) compared the distances between calving sites of woodland caribou in Saskatchewan, Canada, between 2 years to the distribution of distances between the first‐year calving site to all GPS locations used in the following year. Although this approach may control for random movements, comparison of locations of calving sites in year *t* to all locations used the following year would likely inflate distances in the random expectation compared to random locations within a potential calving area in year *t* + 1, which could change conclusions. Using the latter approach, we found that calving‐sequences of caribou expressing spatial fidelity were on average ~ 1.0 km apart, which was considerably less than individuals that did not express spatial fidelity (~15.7 km). This mean distance is closer than previously reported in sedentary woodland caribou in Quebec and Labrador (6.7 km, Schaefer et al., 2000), in woodland caribou in the central mountains of British Columbia and Alberta, Canada (8.7 km, Nobert et al., 2016), and in Svalbard reindeer (*R. t. platyrhynchus*, 1.5–3.9 km, Garfelt‐Paulsen et al., 2021). In fact, three caribou observed in this study calved within 50 m of the site where they calved the previous year.

The variation in caribou expressing spatial fidelity was not related to habitat quality as we had hypothesized, but to age. We recognize there may be error in age estimates when using tooth wear and eruption (van den Berg et al., 2021), but we would not expect a systematic bias for fidelity type. Indeed, we still found older individuals were more likely to express spatial fidelity after adding a random 2‐year error into the age analysis. Studies have reported that spatial fidelity in birds is related to age (Beletsky & Orians, 1987; Harvey et al., 1984; Pyle et al., 2001), but other evidence for ungulates is limited. Morrison et al. (2021) did not find that spatial fidelity was related to age in 205 elk (*Cervus canadensis*), 80 moose, 167 mule deer (*Odocoileus hemionus*), or 81 pronghorn. However, their conclusions were based on Euclidean distance calculated between GPS locations on each Julian day between year *t* and year *t* + 1 and not for a specific life history event like calving. Age may reflect the accumulation of experience and greater familiarity with how environmental conditions influence reproductive outcomes (Fagan et al., 2013). On the other hand, an increase in spatial fidelity with age may support the terminal investment hypothesis (Williams, 1966), if spatial fidelity promotes higher fitness, such as calf survival.

We did not find evidence to support that caribou expressing spatial fidelity led to higher calf survival with our small sample of only 27 caribou. A link between juvenile survival and site fidelity was observed in pronghorn (Wiseman et al., 2006), but there were mixed results in moose (McLaren & Patterson, 2021; Testa et al., 2000; Welch et al., 2000). Lafontaine et al. (2017) using a slightly larger sample size (*n* = 33) in Quebec, Canada documented that caribou were more likely to express spatial fidelity during calving if they did not lose their calf the previous year, which corresponds with the “win‐stay: lose‐switch” strategy (Switzer, 1993). Considering spatial fidelity as a binary behavior, compared to varying strengths of spatial fidelity (e.g., Faille et al. 2010, Morrison et al., 2021), may also have limited our capacity to detect an effect of calf survival. We found only 2 of the 19 caribou that we monitored for greater than 2 years expressed spatial fidelity in all years, suggesting behavioral plasticity in expressing spatial fidelity.

We were unable to evaluate the relationship between habitat predictability and spatial fidelity and habitat quality given a lack of variation in values of habitat predictability. In contrast, studies in Quebec, Canada, have documented that caribou express greater spatial fidelity in regions with higher habitat fragmentation (Lafontaine et al., 2017) and natural disturbances (i.e., forest fires; Faille et al., 2010). Our focus on habitat predictability within the pre‐calving‐neonatal range versus a broader area may have limited the variation in values of habitat predictability observed and may not represent a sufficient temporal period to evaluate the predictability of habitats given disturbance regimes. Even if caribou based their expectations of predictability on changes over the full year and across several years, only 0.004–2% of any of our three study regions were cumulatively disturbed by fire and timber harvest over the 5‐year period of this study. Therefore, where disturbance events are rare, long‐term studies with greater spatial or temporal sampling frameworks may be needed to address hypotheses of how environmental predictability influences the propensity of expressing fidelity.

### 5.2 Habitat fidelity

Compared to spatial fidelity, we found half of the calving‐sequences of caribou expressed habitat fidelity, suggesting this may be an important strategy during the key neonatal period. In fact, 7 of the 19 caribou monitored for greater than 2 years expressed habitat fidelity in all years. Six of these seven caribou used predominately lowland conifer forests across all years with one individual preferentially using upland conifer forests across years. We predicted caribou would express habitat fidelity and specialize in high‐quality habitats when they were abundantly available because of low search time (Charnov, 1976; MacArthur & Pianka, 1966). This change in habitat use (or selection) as a function of changing availability is also known as a functional response in habitat use (or selection; Holbrook et al., 2019; Mysterud & Ims, 1998). In contrast, individuals would expand their selection for lower habitat quality when higher‐quality habitats are scarce because of the greater costs associated with finding high‐quality habitats. However, in our study, the probability of expressing habitat fidelity increased as the availability of high‐quality habitat was low. This may reflect caribou becoming “obligate specialist” with a narrow realized niche as the availability of high‐quality habitat declines (Shipley et al., 2009). Characterizing habitat quality based on more than land cover types and linear anthropogenic feature density may also provide a more mechanistic understanding of the relationship between availability of high‐quality habitat and expressing habitat fidelity.

We found higher values of habitat quality, as predicted via an RSF, reflected caribou selecting for upland and lowland conifer forests void of linear anthropogenic features during calving, which are patterns previously documented across Ontario during calving and throughout the year (Hornseth & Rempel, 2016; Viejou et al., 2018; Walker et al., 2021). The RSF also had an k‐fold cross‐validation score of 0.80, suggesting some individual differences in selection patterns. This likely reflects differences between individuals using predominately uplands or lowland conifer forests during the neonatal period, e.g., 74% and 22% of percent of calving‐sequences that expressed habitat fidelity used predominately lowland and upland conifer forests, respectively. Upland conifer forests may indirectly reduce predation risk due to spatial segregation from alternative prey, i.e., moose, which select for mixedwood and deciduous forests (Bowman et al., 2010; Street et al., 2015), and selection for lowland conifer forest during calving may reflect direct avoidance of predation risk from bears and wolves that select against these areas (Kittle et al., 2015, 2017; Latham et al., 2011; McLoughlin et al., 2005; Mosnier et al., 2008). Thus, avoiding predation may explain why caribou do not generalize habitat use during calving and are instead consistent in the habitat characteristics they select. However, we could not link the consistent selection for these types of habitat characteristics to calf survival, but selection patterns, in general, have been related to fitness consequences (Gaillard et al., 2010; McLoughlin et al., 2006).

### 5.3 Conservation implications

Emphasis on identifying fidelity during calving has been a priority to help guide forest management for the persistence of caribou populations by protecting calving areas (Faille et al., 2010). In Ontario, where boreal woodland caribou are listed as “Threatened” (Government of Canada, 2019; Government of Ontario, 2007) the protection of spatial locations associated with caribou calving is outlined in the provincial forest management guidelines (OMNRF, 2014b; Racey et al., 1999). Across northern Ontario, we found more caribou expressed habitat fidelity than spatial fidelity, indicating that protecting not only specific sites but preferred calving habitat may be essential for enhancing opportunities for caribou calving. However, given caribou propensity to space‐out from conspecifics during the calving period as a predator avoidance strategy (Bergerud & Page, 1987; DeMars et al., 2016), it is likely necessary to protect large extents of calving habitat. Because the site characteristics of these calving areas (i.e., upland and lowland conifer forests without linear features) are also selected by caribou throughout the year (Avgar et al., 2015; Courbin et al., 2009; Hornseth & Rempel, 2016; Leblond et al., 2013; Stuart‐Smith et al., 1997), the protection of calving habitat across large extents will also likely provide benefit to caribou outside the calving period.

The relative importance of management priorities for protecting calving habitat will ultimately depend on their association with reproductive success. This requires obtaining sufficient data to thoroughly assess the relationship between calving strategies and their effects on fitness. Merkle et al. (2022) has argued that spatial fidelity may be maladaptive in regions of high anthropogenic disturbances, due to degradation of habitat quality, which results in a fitness cost associated with expressing spatial fidelity, which Dussault et al. (2012) also suggested for caribou in Quebec, Canada. Caribou inhabiting regions with extensive disturbance may also have restricted space use (Beauchesne et al., 2014) and therefore result in a higher probability of expressing site fidelity given limited calving options. Consequently, this may result in a non‐uniform (i.e., clumped) distribution of caribou across the landscape making them more predictable to predators (DeMars et al., 2016; Fortin et al., 2013). If expressing spatial fidelity is maladaptive, management options may be limited, and intensive management such as augmenting calf survival with maternal penning may be necessary for small populations (Serrouya et al., 2019).

On the other hand, our results suggest considerable behavioral plasticity in space use during calving both within and among individual caribou, which may reflect the pressure of natural selection for alternative tactics, as occurs in other behaviors such as partial migration (Chapman et al., 2011). Given immediate conservation concerns for this species, we advocate for broadening the management approach to provide sufficient calving habitat, which includes large extents of upland and lowland conifer forests without linear features. We also submit that additional studies are needed to focus on the fitness costs associated with expressing different types of fidelity (spatial fidelity see Lafontaine et al., 2017), which is vital information for the long‐term management of caribou.

## AUTHOR CONTRIBUTIONS

**P. D. Walker:** Conceptualization (lead); formal analysis (lead); investigation (lead); methodology (lead); visualization (lead); writing – original draft (lead); writing – review and editing (equal). **A. R. Rodgers:** Conceptualization (supporting); data curation (equal); funding acquisition (equal); investigation (supporting); writing – review and editing (equal). **J. Shuter:** Data curation (equal); writing – review and editing (equal). **J. M. Fryxell:** Data curation (equal); funding acquisition (equal); writing – review and editing (equal). **E. H. Merrill:** Conceptualization (supporting); formal analysis (supporting); funding acquisition (equal); investigation (supporting); methodology (supporting); supervision (lead); visualization (supporting); writing – original draft (supporting); writing – review and editing (equal).

## CONFLICT OF INTEREST STATEMENT

The authors declare that they have no conflict of interests.

## Supporting information


Data S1.

## Data Availability

Non‐migratory woodland caribou are designated as a threatened species in Ontario, similar to many other regions across Canada. Given caribou can exhibit spatial fidelity to calving areas, as shown in this manuscript, they are considered particularly vulnerable to hunting or other forms of anthropogenic disturbance. Therefore, public access to GPS‐telemetry locations is made available only through request and screening by the Ontario Ministry of Natural Resources and Forestry Natural Heritage Information Centre.
