# Supplementary material for: Woodland caribou calving fidelity: Spatial location, habitat, or both?
Source: Ecol Evol. 2024 May 30;14(6):e11480. doi: 10.1002/ece3.11480 (PMC11139972; doi:10.1002/ece3.11480)
Supplement: Supplementary file 1 — Data S1. [file ECE3-14-e11480-s001.docx]

**Supporting Information for:**

**Woodland caribou calving fidelity: spatial location, habitat, or both?**

Walker, P.D., Rodgers, A.R, Shuter, J., Fryxell, J.M., Merrill, E.H.

Table S1. Accuracy of DeMars approach (DeMars et al. 2013) to predict parturition events of caribou in northern Ontario, Canada, 2010–2014, compared to video-collar footage for 22 video-collared caribou-years, when using fix rate intervals from 2.5 or 3 hrs to 12.5 or 13 hrs. One-hr fixes were rarified to 3 hrs and 13 hrs to be similar to the 2.5-hrs and 13-hrs fix rate interval, respectively, after removing the top 2% of step lengths (as per Walker et al. 2021).

| Fix rate interval | Parturition event | Calf predicted | No calf predicted |
| --- | --- | --- | --- |
| 2.5 or 3-hrs | 100% (22 of 22) | 100% (17 of 17) | 100% (5 of 5) |
| 5-hrs | 100% (22 of 22) | 100% (17 of 17) | 100% (5 of 5) |
| 12.5 or 13-hrs | 91% (20 of 22) | 88% (15 of 17) | 100% (5 of 5) |

Table S2. Spatial and habitat fidelity analyses for the 99 (98 for behavioural syndrome) calving-sequences across northern Ontario, Canada, 2010–2014, where we identified spatial fidelity by comparing the Euclidean distance between the proportion (prop.) of random locations less than or equal to the distance between centroids, and identified habitat fidelity where a logistic regression was fit using the Far North Land Cover types (upland conifer forest, lowland conifer forest, early-seral forest, and mixed-deciduous forest) used at neonatal locations compared to a null model (intercept only). Included is the caribou ID, study regions, year one and two, the use of only lowlands or conifer in both years (in which cases a logistic model was not fit), if the calving-sequence expressed habitat fidelity (based on ΔAIC*_c_* > 4 from the null model), and the dominant land cover used at neonatal locations if the calving-sequence expressed habitat fidelity (NA indicates no dominant land cover, because the calving-sequence was classified as not expressing a behavioural syndrome).

| Caribou ID | Study Region | Year 1 | Year 2 | Spatial fidelity | | Habitat fidelity | | | | | | |
| --- | --- | --- | --- | --- | --- | --- | --- | --- | --- | --- | --- | --- |
|  |  |  |  | Distance | Prop. | Lowland only | Upland conifer only | Habitat fidelity | Habitat AIC*_c_* | Null AIC*_c_* | ΔAIC*_c_* | Dominant land cover |
| CAU151 | Nakina | 2011 | 2012 | 10772.96 | 0.34 | no | no | yes | 94.67 | 94.27 | 0.4 | lowland |
| CAU153 | Nakina | 2010 | 2011 | 2650.09 | 0.21 | no | no | no | 36.55 | 138.08 | 101.53 | NA |
| CAU153 | Nakina | 2010 | 2012 | 2992.81 | 0.04 | no | no | no | 149.37 | 171.3 | 21.93 | NA |
| CAU153 | Nakina | 2011 | 2012 | 643.47 | 0.01 | no | no | no | 66.23 | 115.43 | 49.2 | NA |
| CAU252 | Nakina | 2012 | 2013 | 11227.35 | 0.17 | no | no | no | 48.78 | 70.87 | 22.09 | NA |
| CAU253 | Nakina | 2011 | 2012 | 34814.55 | 0.17 | no | no | yes | 33.99 | 32.6 | 1.39 | lowland/ conifer |
| CAU259 | Nakina | 2011 | 2012 | 11275.20 | 0.73 | no | no | yes | 61.56 | 59.98 | 1.57 | conifer |
| CAU259 | Nakina | 2011 | 2014 | 3065.57 | 0.47 | no | no | no | 15.11 | 157.73 | 142.62 | NA |
| CAU259 | Nakina | 2012 | 2014 | 13909.48 | 0.73 | no | no | no | 4.12 | 80.14 | 76.02 | NA |
| CAU263 | Nakina | 2011 | 2012 | 6045.18 | 0.80 | no | no | yes | 120.45 | 123.86 | 3.4 | lowland |
| CAU268 | Nakina | 2011 | 2012 | 7434.32 | 0.73 | no | no | no | 281.74 | 299.39 | 17.65 | NA |
| CAU268 | Nakina | 2011 | 2013 | 9539.38 | 0.32 | no | no | no | 242.61 | 263.77 | 21.16 | NA |
| CAU268 | Nakina | 2012 | 2013 | 2509.19 | 0.04 | no | no | no | 377.02 | 385.56 | 8.54 | NA |
| CAU269 | Nakina | 2011 | 2012 | 4056.14 | 0.22 | no | no | no | 92.07 | 105.99 | 13.92 | NA |
| CAU269 | Nakina | 2011 | 2013 | 24027.53 | 0.27 | no | no | no | 107.21 | 153.48 | 46.27 | NA |
| CAU269 | Nakina | 2011 | 2014 | 26044.67 | 0.72 | no | no | no | 54.54 | 72.7 | 18.16 | NA |
| CAU269 | Nakina | 2012 | 2013 | 7800.71 | 0.06 | no | no | yes | 69.44 | 70 | 0.56 | lowland |
| CAU269 | Nakina | 2012 | 2014 | 28447.83 | 0.75 | no | no | yes | 38.8 | 40.64 | 1.84 | lowland |
| CAU269 | Nakina | 2013 | 2014 | 27265.31 | 0.62 | no | no | yes | 53.62 | 52.7 | 0.93 | lowland |
| CAU272 | Nakina | 2011 | 2012 | 132.68 | 0.00 | no | no | yes | 180.31 | 180.28 | 0.03 | conifer |

Table S2. Continued.

| Caribou ID | Study Region | Year 1 | Year 2 | Spatial fidelity | | Habitat fidelity | | | | | | |
| --- | --- | --- | --- | --- | --- | --- | --- | --- | --- | --- | --- | --- |
|  |  |  |  | Distance | Prop. | Lowlands only | Conifer only | Habitat fidelity | Habitat AIC*_c_* | Distance | Prop. | Dominant land cover |
| CAU273 | Nakina | 2011 | 2012 | 5632.43 | 0.16 | no | no | no | 56.78 | 63.62 | 6.84 | NA |
| CAU273 | Nakina | 2011 | 2013 | 14793.36 | 0.63 | no | no | no | 119.3 | 187.51 | 68.21 | NA |
| CAU273 | Nakina | 2012 | 2013 | 9181.92 | 0.16 | no | no | yes | 56.73 | 59.48 | 2.75 | lowland/ conifer |
| CAU280 | Nakina | 2011 | 2012 | 2615.07 | 0.81 | no | no | yes | 228.46 | 230.93 | 2.47 | lowland |
| CAU281 | Nakina | 2011 | 2012 | 423.83 | 0.07 | no | no | no | 354.7 | 389.65 | 34.95 | NA |
| CAU283 | Nakina | 2011 | 2012 | 15466.34 | 0.72 | no | no | no | 257.03 | 331.53 | 74.5 | NA |
| CAU285 | Nakina | 2011 | 2012 | 2707.54 | 0.64 | no | no | no | 33.63 | 120.14 | 86.52 | NA |
| CAU296 | Nakina | 2011 | 2012 | 1059.41 | 0.14 | no | no | yes | 356.82 | 357.64 | 0.81 | conifer |
| CAU296 | Nakina | 2011 | 2013 | 198.21 | 0.00 | no | no | yes | 237.47 | 234.99 | 2.48 | conifer |
| CAU296 | Nakina | 2012 | 2013 | 947.63 | 0.11 | no | no | yes | 242.29 | 245.09 | 2.8 | conifer |
| CAU297 | Nakina | 2011 | 2012 | 3504.93 | 0.15 | no | no | no | 6.17 | 187.53 | 181.36 | NA |
| CAU297 | Nakina | 2011 | 2013 | 3706.00 | 0.09 | no | no | no | 72.95 | 163.28 | 90.33 | NA |
| CAU297 | Nakina | 2012 | 2013 | 513.34 | 0.00 | no | no | no | 192.95 | 228.06 | 35.11 | NA |
| CAU312 | Nakina | 2012 | 2013 | 1543.12 | 0.22 | no | no | no | 635.93 | 721.72 | 85.8 | NA |
| CAU314 | Nakina | 2012 | 2013 | 35203.22 | 0.77 | no | no | no | 228.72 | 268.63 | 39.91 | NA |
| CCO180 | Cochrane | 2010 | 2011 | 79.00 | 0.00 | yes | no | yes | NA | NA | NA | lowland |
| CCO180 | Cochrane | 2010 | 2012 | 2645.89 | 0.10 | yes | no | yes | NA | NA | NA | lowland |
| CCO180 | Cochrane | 2010 | 2013 | 3093.55 | 0.25 | yes | no | yes | NA | NA | NA | lowland |
| CCO180 | Cochrane | 2011 | 2012 | 481.57 | 0.01 | yes | no | yes | NA | NA | NA | lowland |
| CCO180 | Cochrane | 2011 | 2013 | 2567.10 | 0.11 | yes | no | yes | NA | NA | NA | lowland |
| CCO180 | Cochrane | 2012 | 2013 | 3014.55 | 0.24 | yes | no | yes | NA | NA | NA | lowland |
| CCO209 | Cochrane | 2011 | 2012 | 1770.77 | 0.01 | no | no | yes | 133.91 | 134.49 | 0.58 | lowland |
| CCO209 | Cochrane | 2011 | 2013 | 9929.92 | 0.17 | no | no | yes | 218.6 | 222.06 | 3.46 | lowland |
| CCO209 | Cochrane | 2012 | 2013 | 9667.59 | 0.16 | yes | no | yes | NA | NA | NA | lowland |
| CCO210 | Cochrane | 2011 | 2012 | 46363.23 | 0.77 | no | no | no | 95.42 | 105.86 | 10.44 | NA |
| CCO212 | Cochrane | 2011 | 2012 | 101.91 | 0.00 | no | no | yes | 292.5 | 290.95 | 1.56 | lowland |
| CCO214 | Cochrane | 2011 | 2012 | 280.18 | 0.00 | no | no | yes | 78.67 | 78.16 | 0.51 | lowland |

Table S2. Continued.

| Caribou ID | Study Region | Year 1 | Year 2 | Spatial fidelity | | Habitat fidelity | | | | | | |
| --- | --- | --- | --- | --- | --- | --- | --- | --- | --- | --- | --- | --- |
|  |  |  |  | Distance | Prop. | Lowlands only | Conifer only | Habitat fidelity | Habitat AIC*_c_* | Distance | Prop. | Dominant land cover |
| CCO214 | Cochrane | 2011 | 2013 | 281.84 | 0.00 | no | no | yes | 153.51 | 155.44 | 1.93 | lowland |
| CCO214 | Cochrane | 2012 | 2013 | 25.74 | 0.00 | no | no | yes | 146.66 | 144.74 | 1.93 | lowland |
| CCO215 | Cochrane | 2011 | 2012 | 16269.55 | 0.69 | no | no | no | 144.71 | 149.98 | 5.27 | NA |
| CCO219 | Cochrane | 2011 | 2012 | 8558.31 | 0.21 | no | no | no | 100.13 | 119.62 | 19.49 | NA |
| CCO221 | Cochrane | 2011 | 2012 | 2908.50 | 0.13 | no | no | no | 193.53 | 203.99 | 10.45 | NA |
| CCO223 | Cochrane | 2011 | 2012 | 4043.79 | 0.80 | no | no | yes | 359.51 | 361.99 | 2.48 | lowland |
| CCO223 | Cochrane | 2011 | 2013 | 4113.91 | 0.52 | yes | no | yes | NA | NA | NA | lowland |
| CCO223 | Cochrane | 2012 | 2013 | 156.68 | 0.00 | no | no | yes | 232.65 | 232.99 | 0.34 | lowland |
| CCO224 | Cochrane | 2011 | 2012 | 15520.48 | 0.06 | no | no | yes | 84 | 81.85 | 2.15 | lowland |
| CCO225 | Cochrane | 2011 | 2012 | 377.78 | 0.00 | no | no | no | 197.42 | 216.02 | 18.6 | NA |
| CCO225 | Cochrane | 2011 | 2013 | 1242.85 | 0.01 | no | no | no | 128.85 | 135.97 | 7.13 | NA |
| CCO225 | Cochrane | 2012 | 2013 | 1047.58 | 0.00 | no | no | no | 69.3 | 95.31 | 26 | NA |
| CCO226 | Cochrane | 2011 | 2012 | 39350.08 | 0.79 | no | no | no | 197.47 | 253.4 | 55.93 | NA |
| CCO230 | Cochrane | 2011 | 2012 | 1060.92 | 0.00 | yes | no | yes | NA | NA | NA | lowland |
| CCO233 | Cochrane | 2011 | 2012 | 3276.48 | 0.08 | yes | no | yes | NA | NA | NA | lowland |
| CCO233 | Cochrane | 2011 | 2013 | 19232.47 | 0.81 | yes | no | yes | NA | NA | NA | lowland |
| CCO233 | Cochrane | 2012 | 2013 | 22364.00 | 0.73 | yes | no | yes | NA | NA | NA | lowland |
| CCO234 | Cochrane | 2011 | 2012 | 15687.54 | 0.83 | yes | no | yes | NA | NA | NA | lowland |
| CCO234 | Cochrane | 2011 | 2013 | 37748.85 | 0.66 | yes | no | yes | NA | NA | NA | lowland |
| CCO234 | Cochrane | 2012 | 2013 | 42464.06 | 0.85 | yes | no | yes | NA | NA | NA | lowland |
| CCO235 | Cochrane | 2012 | 2013 | 14084.36 | 0.17 | no | no | no | 118.84 | 154.56 | 35.72 | NA |
| CCO235 | Cochrane | 2012 | 2014 | 12485.64 | 0.15 | no | no | yes | 208.01 | 207.34 | 0.66 | lowland |
| CCO235 | Cochrane | 2013 | 2014 | 4573.00 | 0.04 | no | no | no | 61.51 | 83.84 | 22.33 | NA |
| CCO236 | Cochrane | 2011 | 2013 | 4425.84 | 0.00 | no | no | no | 40.59 | 77.58 | 36.99 | NA |
| CCO237 | Cochrane | 2011 | 2012 | 1572.52 | 0.00 | no | no | no | 4.31 | 28.52 | 24.21 | NA |
| CCO239 | Cochrane | 2011 | 2012 | 21388.84 | 0.25 | yes | no | yes | NA | NA | NA | lowland |
| CCO239 | Cochrane | 2011 | 2013 | 2306.67 | 0.52 | no | no | no | 70.45 | 88.36 | 17.91 | NA |

Table S2. Continued.

| Caribou ID | Study Region | Year 1 | Year 2 | Spatial fidelity | | Habitat fidelity | | | | | | | |
| --- | --- | --- | --- | --- | --- | --- | --- | --- | --- | --- | --- | --- | --- |
|  |  |  |  | Distance | Prop. | Lowlands only | Conifer only | Habitat fidelity | Habitat AIC*_c_* | Distance | Prop. | Dominant land cover |  |
| CCO240 | Cochrane | 2011 | 2012 | 343.21 | 0.02 | NA | NA | NA | NA | NA | NA | NA |  |
| CCO305 | Cochrane | 2012 | 2013 | 42195.50 | 0.26 | yes | no | yes | NA | NA | NA | lowland |  |
| CPL102 | Pickle Lake | 2010 | 2011 | 14212.91 | 0.15 | no | no | no | 88.49 | 124.24 | 35.74 | NA |  |
| CPL103 | Pickle Lake | 2011 | 2013 | 31.26 | 0.00 | no | no | no | 187.97 | 193.72 | 5.75 | NA |  |
| CPL104 | Pickle Lake | 2010 | 2012 | 11437.95 | 0.40 | no | no | no | 48.24 | 124.48 | 76.24 | NA |  |
| CPL104 | Pickle Lake | 2010 | 2013 | 2474.34 | 0.02 | no | no | no | 38.87 | 130.74 | 91.87 | NA |  |
| CPL104 | Pickle Lake | 2012 | 2013 | 12054.64 | 0.32 | no | no | yes | 361.27 | 360.91 | 0.36 | lowland |  |
| CPL105 | Pickle Lake | 2010 | 2011 | 12895.77 | 0.14 | no | no | no | 86.09 | 97.6 | 11.51 | NA |  |
| CPL112 | Pickle Lake | 2010 | 2011 | 36378.58 | 0.71 | no | no | no | 32.71 | 72.29 | 39.58 | NA |  |
| CPL112 | Pickle Lake | 2010 | 2012 | 36921.15 | 0.46 | no | no | yes | 38.61 | 42.32 | 3.71 | lowland/ conifer |  |
| CPL112 | Pickle Lake | 2011 | 2012 | 726.93 | 0.04 | no | no | no | 58.26 | 69.31 | 11.04 | NA |  |
| CPL113 | Pickle Lake | 2011 | 2012 | 15841.94 | 0.49 | no | no | no | 87.31 | 105.07 | 17.77 | NA |  |
| CPL114 | Pickle Lake | 2010 | 2012 | 3661.34 | 0.09 | no | yes | yes | NA | NA | NA | conifer |  |
| CPL115 | Pickle Lake | 2011 | 2012 | 45867.90 | 0.63 | no | no | no | 51.88 | 75.54 | 23.65 | NA |  |
| CPL117 | Pickle Lake | 2012 | 2013 | 31802.25 | 0.18 | no | no | no | 61.03 | 69.5 | 8.48 | NA |  |
| CPL121 | Pickle Lake | 2010 | 2011 | 25723.46 | 0.22 | no | no | no | 18.04 | 34.84 | 16.8 | NA |  |
| CPL134 | Pickle Lake | 2010 | 2011 | 1498.08 | 0.08 | no | no | yes | 117.41 | 118.88 | 1.46 | lowland |  |
| CPL134 | Pickle Lake | 2010 | 2012 | 769.78 | 0.03 | no | no | no | 15.72 | 82.91 | 67.18 | NA |  |
| CPL134 | Pickle Lake | 2011 | 2012 | 1723.45 | 0.10 | no | no | no | 67.24 | 161.84 | 94.6 | NA |  |
| CPL136 | Pickle Lake | 2010 | 2011 | 1992.83 | 0.07 | no | no | no | 85.11 | 165.69 | 80.58 | NA |  |
| CPL138 | Pickle Lake | 2010 | 2011 | 98135.97 | 0.72 | no | no | yes | 249.71 | 249.33 | 0.39 | conifer |  |
| CPL141 | Pickle Lake | 2012 | 2013 | 3078.52 | 0.06 | no | no | yes | 83.98 | 84.54 | 0.56 | disturbed |  |
| CPL202 | Pickle Lake | 2011 | 2012 | 2235.27 | 0.54 | no | no | no | 4.13 | 131.01 | 126.88 | NA |  |
| CPL203 | Pickle Lake | 2011 | 2012 | 23.81 | 0.00 | no | yes | yes | NA | NA | NA | conifer |  |

Table S3. Number (*n*) of calving-sequences, beta coefficient (β) and confidence interval (CI) from independent, multivariable logistic mixed-effect models predicting the probability of caribou calving-sequences expressing a type of fidelity (1: no fidelity or habitat fidelity [neither], spatial fidelity, habitat fidelity, or both spatial and habitat fidelity) compared to not expressing that fidelity type (0) as a function of habitat quality in the pre-calving-neonatal 95% utilization distribution and caribou age (years) across three study regions in northern Ontario, Canada, based on caribou telemetry data from 2010−2014. Asterix indicates confidence intervals do not overlap zero.

| Fidelity type | *n* | Habitat quality | | Age | |
| --- | --- | --- | --- | --- | --- |
|  |  | β | 95% CI | β | 95% CI |
| Neither | 70 | 4.86 | -0.44, 10.16 | -0.47 | -0.95, 0.02 |
| Spatial | 70 | 0.24 | -3.47, 3.94 | 0.44* | 0.05, 0.82 |
| Habitat | 69 | -10.89 | -22.99, 1.21 | 0.04 | -0.50, 0.57 |
| Both | 69 | -8.94 | -22.40, 4.52 | 0.14 | -0.84, 1.13 |

Table S4. Number (*n*) of calving-sequences, beta coefficient (β) and confidence interval (CI) from independent, multivariable logistic mixed-effect models predicting the probability of caribou calving-sequences expressing a type of fidelity (1: no fidelity or habitat fidelity [neither], spatial fidelity, habitat fidelity, or both spatial and habitat fidelity) compared to not expressing that fidelity type (0) as a function of habitat quality (HQ) in the pre-calving-neonatal 95% utilization distribution, caribou age (years), and the interaction (HQ*Age), across three study regions in northern Ontario, Canada, based on caribou telemetry data from 2010−2014.

| Fidelity type | *n* | Habitat quality | | Age | | HQ*Age | |
| --- | --- | --- | --- | --- | --- | --- | --- |
|  |  | β | 95% CI | β | 95% CI | β | 95% CI |
| Neither | 70 | 3.54 | -13.73, 20.81 | -0.7 | -3.70, 2.30 | 0.25 | -2.88, 3.38 |
| Spatial | 70 | 3.42 | -10.72, 17.57 | 0.94 | 1.25, 3.12 | -0.54 | -2.86, 1.77 |
| Habitat | 69 | 1.60 | -22.12, 25.32 | 2.12 | -2.11, 6.35 | -0.24 | -6.66, 2.18 |
| Both | 69 | 6.58 | -29.74, 42.90 | 2.57 | -3.05, 8.20 | -2.84 | -9.33, 3.65 |


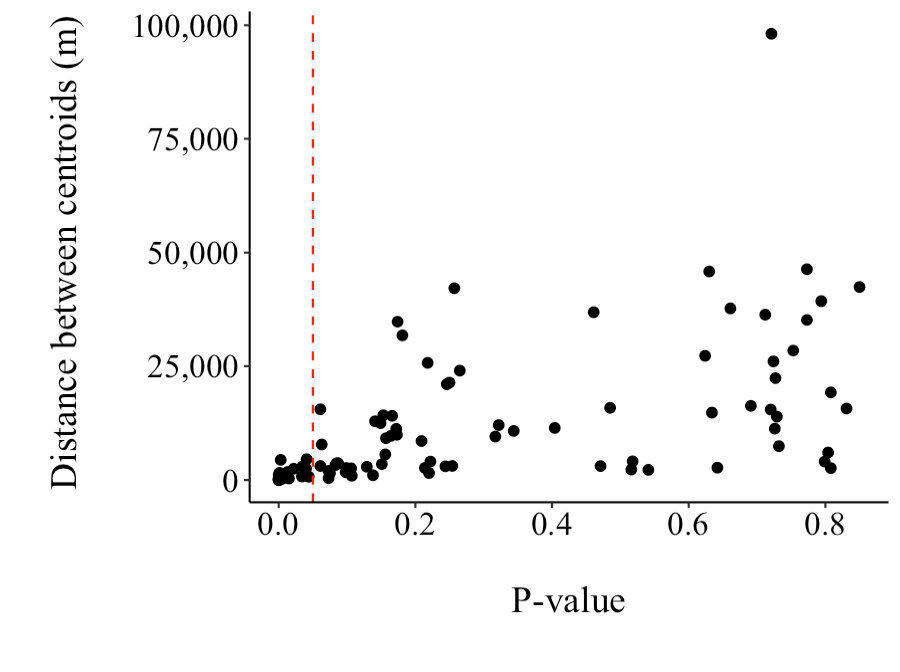


Figure S1. Relationship between the Euclidean distance (m) calculated between neonatal centroid and the p-value (i.e., proportion of random distances less than the Euclidean distance between neonatal centroids) calculated for each calving-sequence. Red-dashed line indicated the p-value threshold of 0.05 used to characterize spatial fidelity, which corresponds to a greater range of Euclidean distances between neonatal centroids with p-values > 0.05 than distances with p-values < 0.05.
